# Supplementary material for: Assessing trade-offs in avian behaviour using remotely collected data from a webcam
Source: PLoS One. 2022 Jul 8;17(7):e0271257. doi: 10.1371/journal.pone.0271257 (PMC9269932; doi:10.1371/journal.pone.0271257)
Supplement: Attachment — Submitted filename: Review of PONE-D-22-04625.docx [file pone.0271257.s001.docx]

Review of “Assessing trade-offs in avian behaviour using remotely collected data from a webcam”

Abstract:

Introduction:

Line 75-83: Following your line of reasoning here that aggression is a means of maintaining access to food resources, I would have hypothesized that birds that were more aggressive had better access to high quality food resources and therefore would need to spend less time feeding as a result.

Line 85-88: These predictions contradict my expectations for why/how aggressive behavior would be rewarded. If a bird that spends a lot of time being aggressive to other birds is rewarded by the richest feeding areas and can quickly obtain the resources it needs then its time spent feeding is less and it receives other benefits like increased survival as feeding can be a risky behavior. Not sure that it is that critical a point as just testing these hypotheses is interesting but the assumption of no correlation between aggression and feeding time stated on Ln 80 is not intuitive.

Methods:

Study System:

Line 96-98: Curious if the migratory whooper swans use the reserve for all of their feeding and roosting needs while in the area or if they predominantly feed or roost somewhere outside the reserve?

Statistical Analyses:

Line168-169: While behaviour may not have varied significantly, I would argue that there was a suggestion of differences between them during different times of day and month – see figure from (13). I would really like to see a breakdown of behaviour by hour from sunrise to sunset to test this.

Results:

Line 176-178: Was it assumed that both species of swans were present on the reserve for the entire day? If not, could the whooper swans be feeding offsite and roosting on the reserve?

Discussion:

Lines 267-268: The disparity could also be due to differences in behavior by time of day between the species. If the more mobile whooper swans are flying out to other areas for various resources, the behaviour observed in the reserve may be biased. If there is movement data associated with the flocks or individuals it would be helpful to present for a clearer picture of their daily behaviour.

Lines 278-295 This is a very nice summary of the limitations of the short duration observations and potential alternative approaches in the future.

Lines 313-317: Given data is collected in 15-minute blocks and the total budget for an individual for a given day would be a much better measure of trade-offs I would caution not to overstate the findings. Does a trade-off during a 15 minute bout have biological (impacts to fitness) for any individual or should those measurements be compared across a greater timescale before we draw conclusions regarding plasticity in behaviour.

Figures and Tables:
